# Transcriptome profiling of human thymic CD4+ and CD8+ T cells compared to primary peripheral T cells

**DOI:** 10.1186/s12864-020-6755-1

**Published:** 2020-05-11

**Authors:** Hanna Helgeland, Ingvild Gabrielsen, Helle Akselsen, Arvind Y. M. Sundaram, Siri Tennebø Flåm, Benedicte Alexandra Lie

**Affiliations:** 1 Department of Medical Genetics, University of Oslo and Oslo University Hospital, 0450 Oslo, Norway; 2 Department of Radiation Biology, Oslo University Hospital, 0379 Oslo, Norway

**Keywords:** RNA-seq, Transcriptome, Human, Thymus, T cells

## Abstract

**Background:**

The thymus is a highly specialized organ of the immune system where T cell precursors develop and differentiate into self-tolerant CD4+ or CD8+ T cells. No studies to date have investigated how the human transcriptome profiles differ, between T cells still residing in the thymus and T cells in the periphery.

**Results:**

We have performed high-throughput RNA sequencing to characterize the transcriptomes of primary single positive (SP) CD4+ and CD8+ T cells from infant thymic tissue, as well as primary CD4+ and CD8+ T cells from infant and adult peripheral blood, to enable the comparisons across tissues and ages. In addition, we have assessed the expression of candidate genes related to autoimmune diseases in thymic CD4+ and CD8+ T cells. The thymic T cells showed the largest number of uniquely expressed genes, suggesting a more diverse transcription in thymic T cells. Comparing T cells of thymic and blood origin, revealed more differentially expressed genes, than between infant and adult blood. Functional enrichment analysis revealed an over-representation of genes involved in cell cycle and replication in thymic T cells, whereas infant blood T cells were dominated by immune related terms. Comparing adult and infant blood T cells, the former was enriched for inflammatory response, cytokine production and biological adhesion, while upregulated genes in infant blood T cells were associated with cell cycle, cell death and gene expression.

**Conclusion:**

This study provides valuable insight into the transcriptomes of the human primary SP T cells still residing within the thymus, and offers a unique comparison to primary blood derived T cells. Interestingly, the majority of autoimmune disease associated genes were expressed in one or more T cell subset, however ~ 11% of these were not expressed in frequently studied adult peripheral blood.

## Background

The thymus is a highly specialized organ of the immune system, where T cell precursors develop and differentiate into self-tolerant single positive (SP) CD4+ or CD8+ T cells, through positive and negative selection [1–3]. No studies, to date, have investigated how the human transcriptome profiles differ between SP T cells still residing in the thymus and T cells in the periphery.

At birth, the majority of peripheral T cells are naïve, consisting mostly of recent thymic emigrants (RTE) (~ 80%) [4]. In the first years of life, the load of microbes and pathogens to be encountered, is at its peak. T cells play a crucial role in protecting the body from these invaders, and due to this antigen exposure, the memory T cells begin to accumulate. The establishment of long-term reserves of memory T cells plateaus at 2nd decade of life, after the involution of the thymus [5]. From ages 1 to 50+, there is a gradual decline of thymic epithelial space [6]. Evidence of ongoing thymopoiesis, measured by signal joint T cell receptor excision circles (sjTREC) levels, show an exponential drop with increasing age, with detectable levels up to age ~ 60 [7, 8]. A recent study suggests that the steepest decline in thymopoiesis occurs at ~ 40 years of age, with a drop in double positive (DP) thymocytes and reduced number of RTEs in lymphoid tissues [9]. This age coincides with the age of onset for many autoimmune diseases.

A high-dimensional atlas of human T cell diversity in eight different tissues has been reported, using CyTOF [10], but neither thymus nor peripheral blood from children was among those tissues. In mice, single-cell transcriptomic atlases of the developing [11] and neonatal murine thymus [12] was recently released, providing detailed insights of the development of thymocytes into mature T cells. Previously, transcriptome profiling using microarray of flow sorted cells from murine thymi has been reported, including for CD4+ and CD8+ T cells [13, 14]. So far, humans studies have explored the gene expression of recent thymic emigrants, immature T cell stages and naïve T cells, derived from peripheral blood [15, 16] and umbilical cord blood [17]. To our knowledge, no one has yet explored the human transcriptome of the finale stage of thymocytes, the SP T cells, or the transcriptome of the peripheral blood T cells in young children.

In this study, we have performed high-throughput RNA sequencing to characterize the transcriptomes of SP CD4+ and CD8+ T cells from primary human thymic tissue, and compared them to CD4+ and CD8+ T cells in infant and adult peripheral blood, providing a unique insight into the mechanisms of T cell migration and differentiation in thymus, infant blood and adult blood.

## Results

### Cell purity and viability assessments

The purity of the CD4+ cells from both tissues was ~ 95% (Supplementary Figure S1–3, Additional File 1). The CD8+ populations displayed more varying purity scores. The thymic CD8+ T cells achieved ~ 95% purity, using negative enrichment. (Supplementary Figure S4, Additional File 1). The positive selection assay for CD8α used on peripheral blood, performed better in adult than infant blood, with purity scores at 95 and 75%, respectively (Supplementary Figure S6–5, Additional File 1). Staining the CD8α + cells after sorting, with CD3 we found that > 90% of the CD8 T cells were CD3+ (Supplementary Figure S7, Additional File 1), suggesting that a small portion of the CD8α + cells could be NK, immature thymocytes or other CD8α + CD3- cells. CD3+ NKT cells may be present, however in supposedly small numbers as NKT cells constitute 1% of all peripheral blood T cells [18]. We detected suspected double positive CD4CD8+ thymocytes in the CD4+ thymocyte population (Supplementary Figure S1, Additional File 1), and vice versa (about 10%) (Supplementary Figure S4, Additional File 1). In the infant blood, we observed 2% CD4+ cells in the CD8+ population (Supplementary Figure S5, Additional File 1), while in adult blood we observed 5% CD4+ cells in the CD8+ population (Supplementary Figure S6, Additional File 1). We also found traces of CD8+ T cells in the isolated CD4+ T cells. This was seen, to a less extent, in CD4+ adult blood (~ 2% CD8+ cells, Supplementary Figure S3, Additional File 1). The viability differed between sample subsets. The thymic samples had a higher average viability (88%) than blood (77%) for CD4+ T cells, while the average viability of CD8+ cells was 63% from thymus and 71% from blood (data not shown).

### Descriptive statistics

**Fig. 1:**
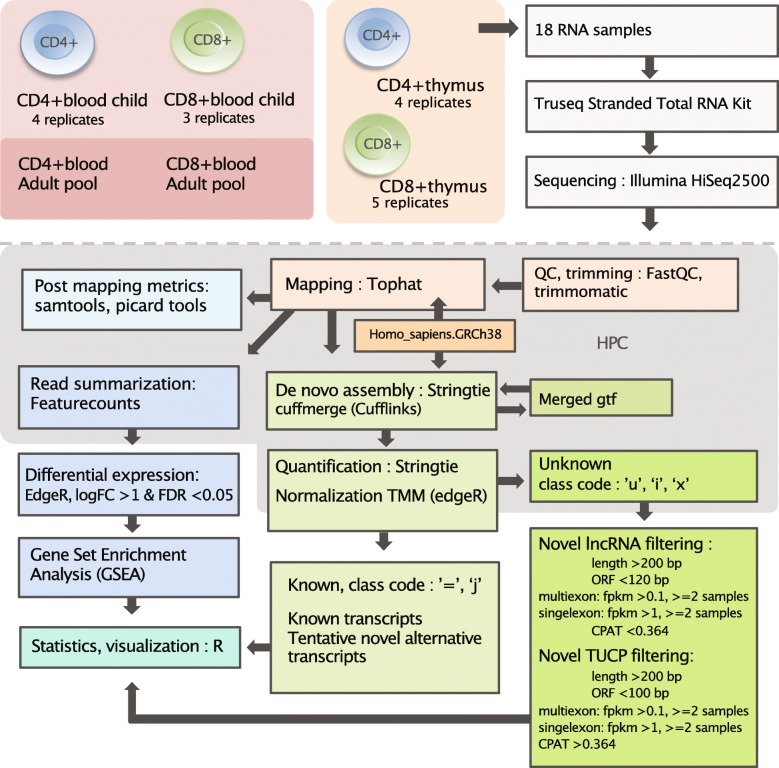
Graphical outline of the experiment

Figure 1 provides a graphical overview of the experimental design and workflow. For the SP CD4+ and CD8+ T cells from infant thymus and blood, we used 3–5 biological replicates (ages 5 days – 15 months), while peripheral blood CD4+ and CD8+ T cells from adults were pooled from five individuals (23–45 years). From all 18 transcriptome profiles generated, the sequencing depth ranged from 69 to 122 M reads (Supplementary Table S1, Additional File 2). However, particularly the sequencing data from the CD8+ T cells contained a considerable proportion of multimapping reads (28–86%). Yet, after excluding multimapping reads from further analysis, satisfactory estimated library sizes for detecting DE genes (> 10 M) [19], remained for 14 out of 18 samples (range: 4–67 M, median: 49 M).

### The thymic and peripheral blood T cell transcriptome

**Fig. 2:**
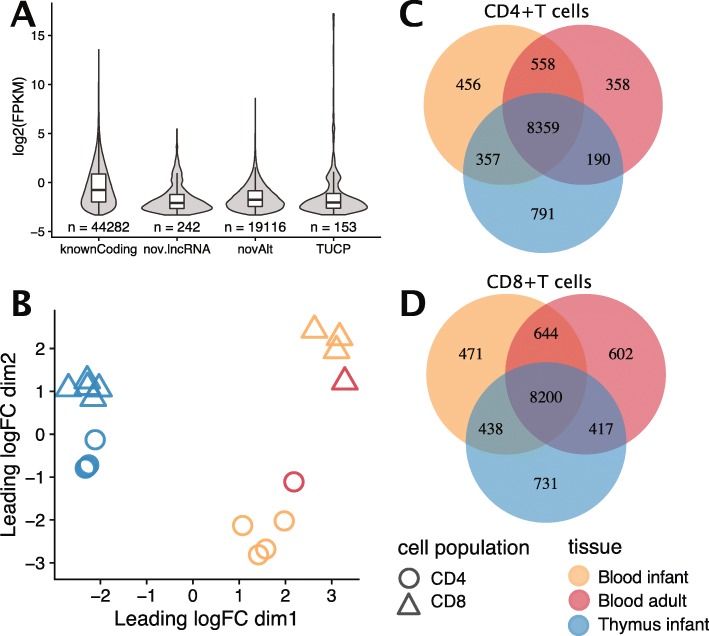
**a** log2 FPKM and total number of known coding transcripts, potentially novel lincRNA, tentative novel alternative transcripts and TUCP (transcript of uncertain coding potential) identified in CD4+ and CD8+ thymic, infant and adult blood derived T cells. **b** MDS plot displaying unsupervised clustering of the samples. The distance corresponds to the average (root-mean-square) of the 500 largest absolute log-fold-changes between each pair of samples. Uniquely and commonly expressed genes between **c** CD4+ and **d** CD8+ thymic, infant and adult blood T cells, at a threshold of FPKM > = 2

RNA-seq of human CD4+ and CD8+ T cells, derived from infant thymus, as well as from infant and adult peripheral blood, detected 44,282 known coding transcripts (Fig. 2a). In addition, 19,116 potentially novel alternative transcripts, 242 novel long non-coding RNA (lncRNA) and 153 novel transcripts of uncertain coding potential (TUCP) were also uncovered. The novel alternative transcripts displayed the largest range in number of exons, with 26.5% of the transcripts exceeding 20 exons (Supplementary Figure S1A, Additional File 3), showed a high coding probability (median 0.99, Supplementary Figure S1B, Additional File 3), and comprised the longest transcripts, with 30% exceeding 10 kb (Supplementary Figure S1C, Additional File 3). The median coding probability was high also for the generally shorter TUCP (0.67), while it was very low (0.004) for the novel lncRNA. Both TUCP and lncRNA had a median of two exons. Investigating thymic SP T cells exclusively, 39,965 known transcripts, 20,764 potentially novel alternative transcripts, 252 potentially novel lncRNA and 171 transcripts of uncertain coding potential (Supplementary Figure S1D, Additional File 3) were detected. Infant CD4+ T cells of blood and thymic origin presented similar numbers of detected transcripts, while for the CD8+ T cells, the infant blood derived displayed ~ 30% less transcripts than the thymic T cells (Table 1). The adult blood derived transcripts were consistently the least abundant.

**Table 1:** Number of known coding transcripts, potentially novel lincRNA, tentative alternative transcripts and TUCP (transcript of uncertain coding potential) identified in CD4+ and CD8+ thymic, infant and adult blood derived T cells

Cell	Group	Known coding	Novel lncRNA	Novel alterntive transcripts	TUCP
CD4	adult blood	31,906	64	9807	35
CD4	infant blood	38,857	107	14,389	56
CD4	infant thymus	37,886	124	11,691	57
CD8	adult blood	18,249	30	5022	29
CD8	infant blood	28,108	65	8366	42
CD8	infant thymus	39,058	139	12,962	109

### Genes expressed in T cells from human thymus and blood

RNA-seq of the primary T cell subsets from human thymus and blood identified transcripts from 18,218 known genes in total, after filtering low expressed genes (< 1 pr million counts) (Supplementary Figure S2, Additional File 3). 14,441 (79%) were protein coding (representing 61% of Ensembl protein coding genes), 2501 lncRNA, 944 pseudogenes and 332 non-coding RNA (ncRNA). A multidimensional scaling (MDS) plot of the transcriptomes (Fig. 2b), revealed that the samples were separated by tissue in the first dimension and by cell type in the second dimension. Both thymic SP CD4+ (Fig. 2c) and CD8+ T cells (Fig. 2d) showed more uniquely expressed genes (average gene expression FPKM> 2 for the replicates) than the blood derived T cells from infants or adults. A higher number of expressed genes were shared between thymic CD4+ and thymic CD8+ T cells, than between infant blood vs thymic T cells of the same cell population (Supplementary Figure S3A, Additional File 3). This pattern was also true for genes associated with autoimmune diseases (Supplementary Figure S3B, Additional File 3).

### Genes associated with autoimmune diseases

**Fig. 3:**
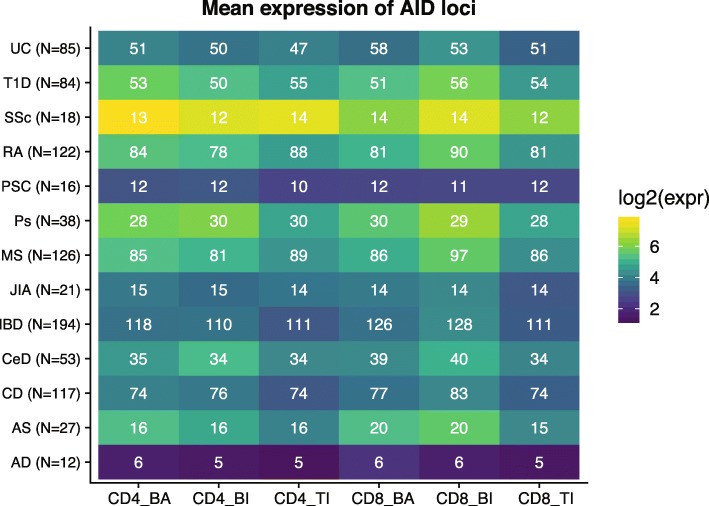
Mean expression (log2 FPKM, visualized by the blue-yellow color scale) and number of genes expressed at FPKM > = 2 (represented by white numbers) of 555 AID associated genes, for each condition and cell population. BA = blood adult, BI = blood infant, TI = thymus infant

Of 555 loci associated with autoimmune diseases (AID; GWAS catalogue Nov 2015, *P* < 5 × 10^− 8^), the majority were expressed in our T cell datasets. Only 123 (22.2%) of the annotated genes were not detected (at FPKM > = 2) in neither CD4+ nor CD8+ T cells from any of the three origins, while more than half of the genes (*N* = 285) were expressed in both T cell populations from all sample types (Supplementary Table S2, Additional File 2). The proportion of AID genes expressed varied across our T cell populations and between the diseases (Fig. 3). For the AIDs we investigated, at least half of the identified risk genes were found to be expressed. Observing the T cell populations separately, 378 of AID associated genes were expressed by CD4+ of any origin and 421 genes were expressed by CD8+ of any origin (Supplementary Figure S3C-D, Additional File 3). Interestingly, 49 of the 432 expressed AID genes were not expressed in T cells from adult blood (Supplementary Table S2, Additional File 2). Of these 18 AID risk genes were only expressed in thymic SP T cells while 20 AID risk genes were only detected in peripheral T cells from children. These 49 loci were mainly associated with inflammatory bowel disease (*N* = 21), multiple sclerosis (*N* = 18), rheumatoid arthritis (*N* = 15) and type 1 diabetes (*N* = 10).

### Differential expression was most pronounced between thymus and blood

In both CD4+ and CD8+ T cells, the largest number of differentially expressed genes (DEGs) was discovered when comparing T cells from thymus with infant blood, followed by adult blood (Table 2). Comparing infant with adult blood T cells provided less DEGs. Similarly, when comparing the transcriptomes of CD4+ with CD8+ T cells, from different origins (Table 2), the highest numbers of DEGs were observed between the two T cell subpopulations in thymus, followed by infant blood, and lastly, adult blood. Volcano plots of DEGs for the pairwise comparisons are shown in Supplementary Figure S4 (Additional File 3), and complete lists of DEGs with expression values for all samples are found in Supplementary Tables S3–11 (Additional File 2).

**Table 2:** Number of significantly differentially expressed genes (DEGs) from the pairwise comparisons, at FDR < 0.05, and additional criteria logCPM> 1.5 and logFC> 1

group	comparison	upregulated in	#DEGs	#DEGs total
CD4+	thymus vs infant blood	thymus	1624	2957
		infant blood	1333	
CD4+	thymus vs adult blood	thymus	1451	2688
		adult blood	1237	
CD4+	infant blood vs adult blood	infant blood	246	575
		adult blood	329	
CD8+	thymus vs infant blood	thymus	1286	2695
		infant blood	1409	
CD8+	thymus vs adult blood	thymus	1154	2222
		adult blood	1068	
CD8+	infant blood vs adult blood	infant blood	250	405
		adult blood	155	
adult blood	CD4+ vs CD8+	CD4+	339	675
		CD8+	336	
infant blood	CD4+ vs CD8+	CD4+	819	1995
		CD8+	1176	
thymus	CD4+ vs CD8+	CD4+	1107	2028
		CD8+	921	

**Fig. 4:**
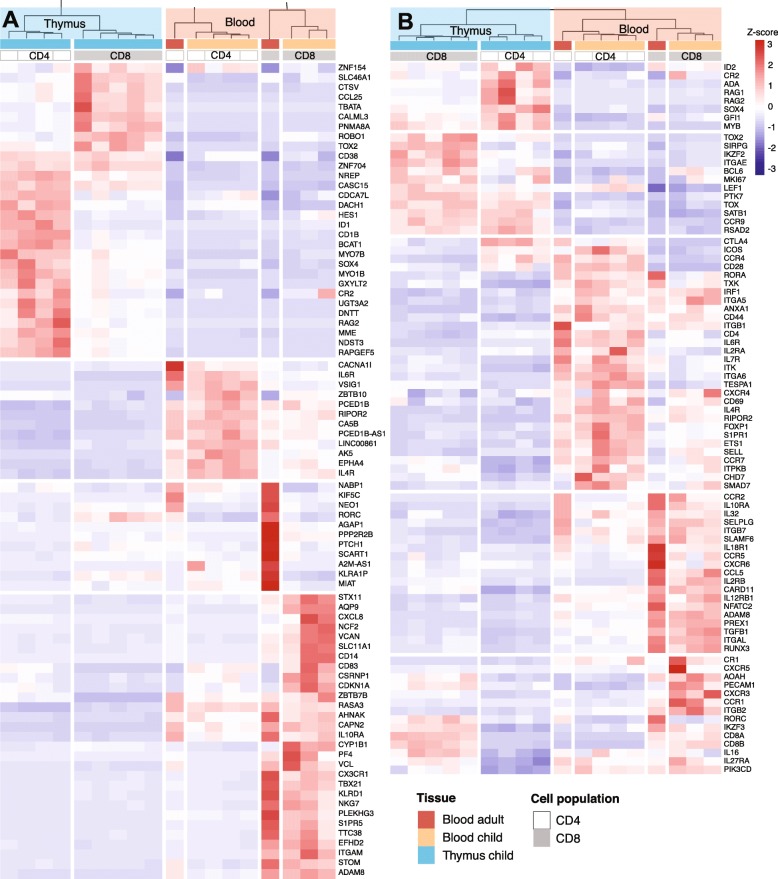
**a** Top 10 up and downregulated genes (FDR < 0.05, logCPM> 1.5, logFC> 1), sorted by FDR, from 6 comparisons; CD4+ thymic vs infant blood, thymic vs adult blood and infant vs adult blood and CD8+ thymic vs infant blood, thymic vs adult blood and infant vs adult blood. **b** Expression patterns of selected DEGs (FDR < 0.05, logCPM> 1.5, logFC> 1) involved in T cell function, development or migration. The color scale represents z-scores

Clustering the, in total, 5925 DEGs from all comparisons, revealed that the subsets clustered according to tissue of origin, then cell type and age – with one major clade for the thymic cells and one major clade for the blood derived cells (Supplementary Figure S5, Additional File 3). Genes associated with V(D) J recombination and T cell commitment, including *RAG2*, *HES1* and *DNTT*, were amongst the top 10 DEGs upregulated in thymic T cells (Fig. 4a). In CD8+ infant and adult blood T cells, the top upregulated genes included genes involved in cell migration and lineage commitment; *S1PR5*, *PLEKHG3*, and *TBX21*, while, amongst others, interleukin receptors *IL6R* and *IL4R* displayed high expression in CD4+ infant and adult peripheral blood T cells.

### Differences in gene set enrichment profiles related to developmental stage

**Fig. 5:**
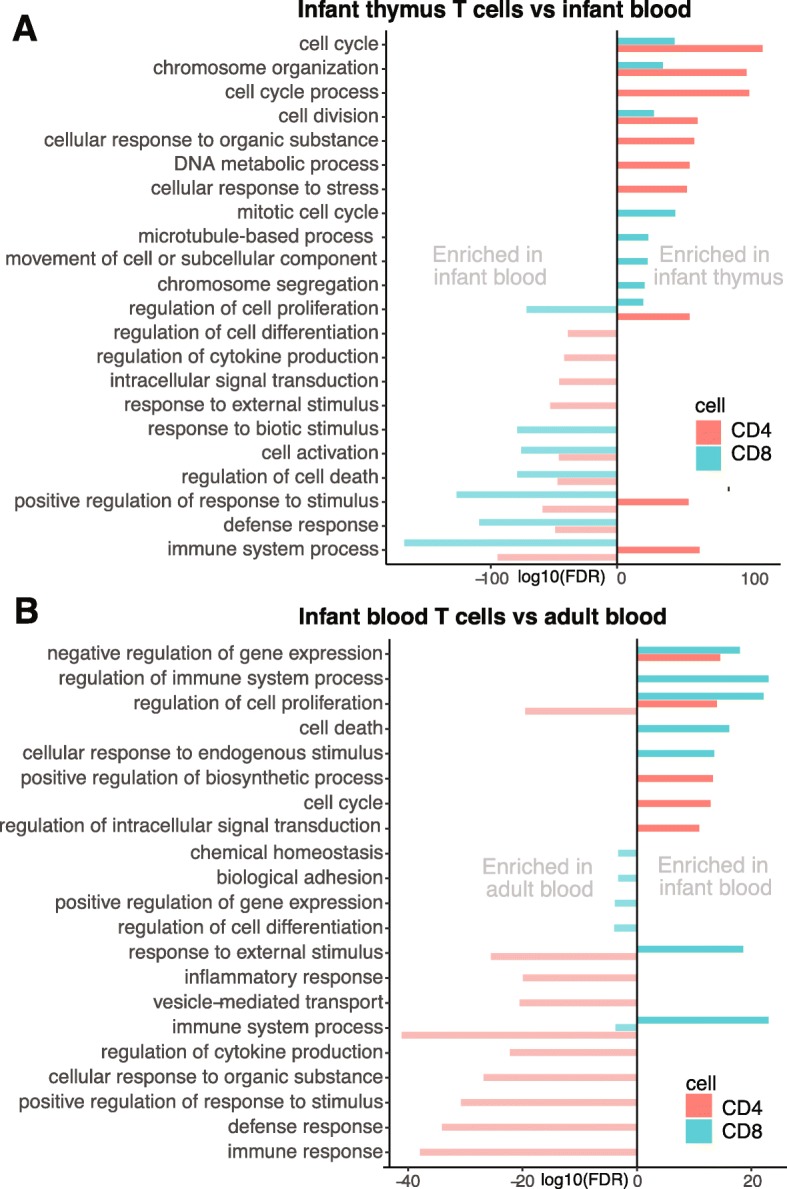
Biological processes enriched when comparing significant DEGs (FDR < 0.05, logCPM> 1.5, logFC> 1) in **a** thymic vs infant blood CD4+ and CD8+ T cells. Negative values represent enriched terms in infant blood, positive values represent enriched terms in infant thymus. **b** Infant vs adult blood CD4+ and CD8+ T cells. Negative values represent enriched terms in adult blood, positive values represent enriched terms in infant blood

The upregulated DEGs in thymic SP CD4+ and CD8+ T cells, were mainly involved in cell division and proliferation, when compared to infant blood CD4+ and CD8+ T cells (Fig. 5a). The DEGs upregulated in infant blood CD4+ and CD8+, compared to the equivalent thymic subset, were enriched for multiple immune related biological processes, such as defense response, cytokine production, and intercellular signal transduction, as well as regulation of cell proliferation and differentiation. When comparing infant to adult blood T cells (Fig. 5b), the infant blood T cells were enriched for genes involved in proliferation and cell death, besides regulation of gene expression and immune system processes. The genes upregulated in adult blood T cells were engaged in response to stimulus, immune and defense response, cytokine production and biological adhesion. Comparing CD4+ to CD8+ T cells, of the same tissue and age, revealed that genes upregulated in thymic CD4+ T cells were heavily involved in chromosome organization and cell cycle, while enriched GO terms in CD8+ T cells in infant blood, were dominated by immune related processes (Supplementary Figure S6, Additional File 3).

### T cell markers for egress, differentiation and migration

**Fig. 6:**
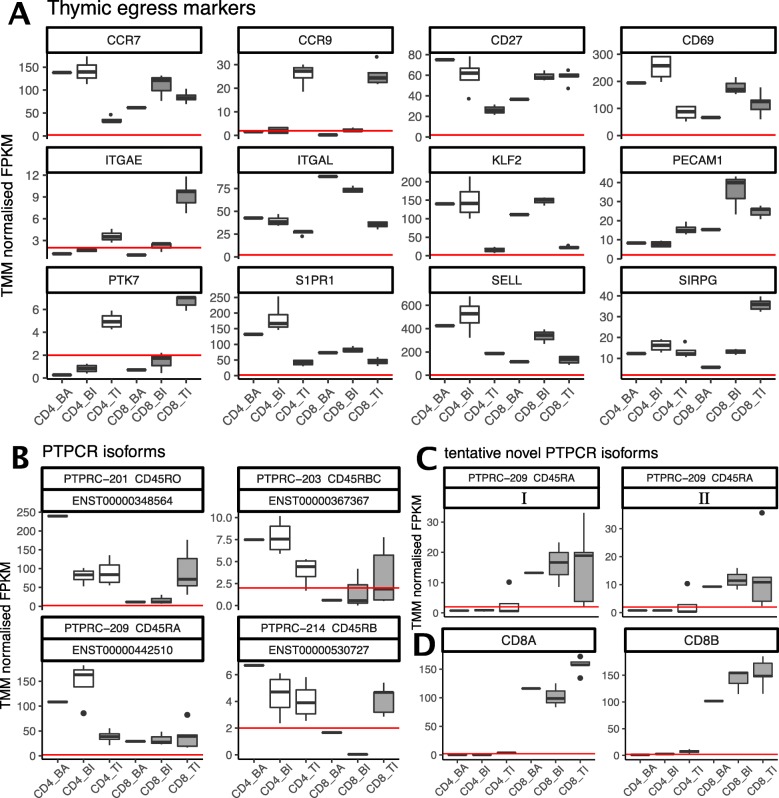
PTPRC (CD45) transcript expression for each T cell subset, for **a** known transcripts and **b** tentative novel isoforms most similar to CD45RABC (PTPRC-209/ ENST00000442510). Gene expression of **c** the CD8A and CD8B genes, and **d** T cell egress markers, for each T cell subset. The horizontal red line represents a cutoff at FPKM = 2. Figure legend abbreviations: BA = blood adult, BI = blood infant, TI = thymus infant

Since we have a unique material of primary T cells from both thymic and blood from infants, we looked specifically at the expression patterns of genes involved in T cell egress (Fig. 6a), migration and differentiation. In general, the CD4+ T cells expressed a wider repertoire of *PTPRC* transcripts than CD8+ T cells (Fig. 6b). In peripheral blood, the adults showed higher expression of CD45RO transcripts (PTPRC-201) in their CD4+ T cells than children, while the opposite was observed for the *CD45RABC* isoform (PTPRC-209). The isoform patterns of CD45 have been less well characterized in CD8+ T cells. We observed tentative novel isoforms (Fig. 6c I and II), sharing exons with *CD45RABC*, in CD8+ T cells, not found to be expressed in CD4+ T cells. In the CD8+ cells, these novel *PTPCR* transcripts were expressed at similar levels as *CD45RABC* and *CD45RO*. We also observed that the *CD45RB* transcripts (PTPRC 203 and 214) displayed higher expression in the peripheral blood CD4+ T cells than the SP CD4+ T cells in the thymus, yet compared to the RO and the RABC isoforms, overall expression was low*.*

We furthermore investigated the CD45RA/RO ratios of the CD4 T cells, at the surface protein level using FACS, comparing a thymic sample and blood from the same child, and blood samples from two adults aged 30 and 70 years (Supplementary Figure S8, Additional File 1). Like others [5, 20], we observed high amounts of CD45RO in the thymic sample, while the blood sample, from the same individual, displayed less CD45RO and more CD45RA positive cells. Both the adult samples, regardless of age, showed extensive co-expression of CD45RA and CD45RO (43–51%, Supplementary Figure S8, Additional File 1), yet the overall expression of CD45RA was low, compared to infant blood. The higher CD45RA expression in infants compared to adults is likely due to a higher proportion of naïve T cells.

Our data suggests that infant CD8+ T cells may express *CD8B* at a higher level than *CD8A*, while the opposite was seen in the adult pool of CD8+ T cells (Fig. 6d), though the difference was not statistically significant. The expression levels of *CD8A* and *CD8B* in the SP thymic T cells were equivalent. We explored the distribution of CD8B isoforms, and detected highest expression of CD8b-201 (ENST00000331469) in SP thymic CD8+ T cells, followed by the blood CD8+ T cells from adults and infants (Supplementary Figure S7, Additional File 3). The most abundant isoform was CD8b-203 (ENST00000390655), mainly expressed by the CD8+ mature thymocytes, followed by the infant blood T cells, and to a lesser degree in adult CD8+ T cells.

To further investigate differentially expressed genes involved in T cell differentiation and migration, we extracted DEGs associated with the GO terms “lymphocyte migration” (GO:0072676) and “T cell differentiation” (GO:0030217), as well as relevant genes from the literature (Fig. 4b). The genes upregulated in thymic T cells included recombination-activating genes; *RAG1* and *RAG2*, genes involved in adhesion and homing; *ITGAE* (CD103) and *CCR9*, T lineage commitment; *SATB1*, cell proliferation; *MKI67* and transcriptional regulators involved in T cell development; *ID2*, *SOX4*, *LEF1* and *BCL6*. In adult blood T cells, several chemokines, interleukins, and their receptors were upregulated; *CCL5* (RANTES), *IL12RB1*, *IL10RA*, *IL32*, *CCR2* and *CCR5*, as well as genes involved in cell adhesion and migration; *ADAM8*, *ITGB7*, *SELPLG*, and lymphocyte function and activation, including *SLAMF6*, *PIK3CD*, *TXK* and *NFATC2*. Several genes involved in cell adhesion and lymphocyte homing, migration, egress and maturation were upregulated in infant blood T cells; *CD69*, *CD44*, *SELL* (*CD62L*), *CCR7*, *S1PR1*, *ITGA6*, *ITGA5*, *ITK* and *TESPA1*.

## Discussion

**Fig. 7:**
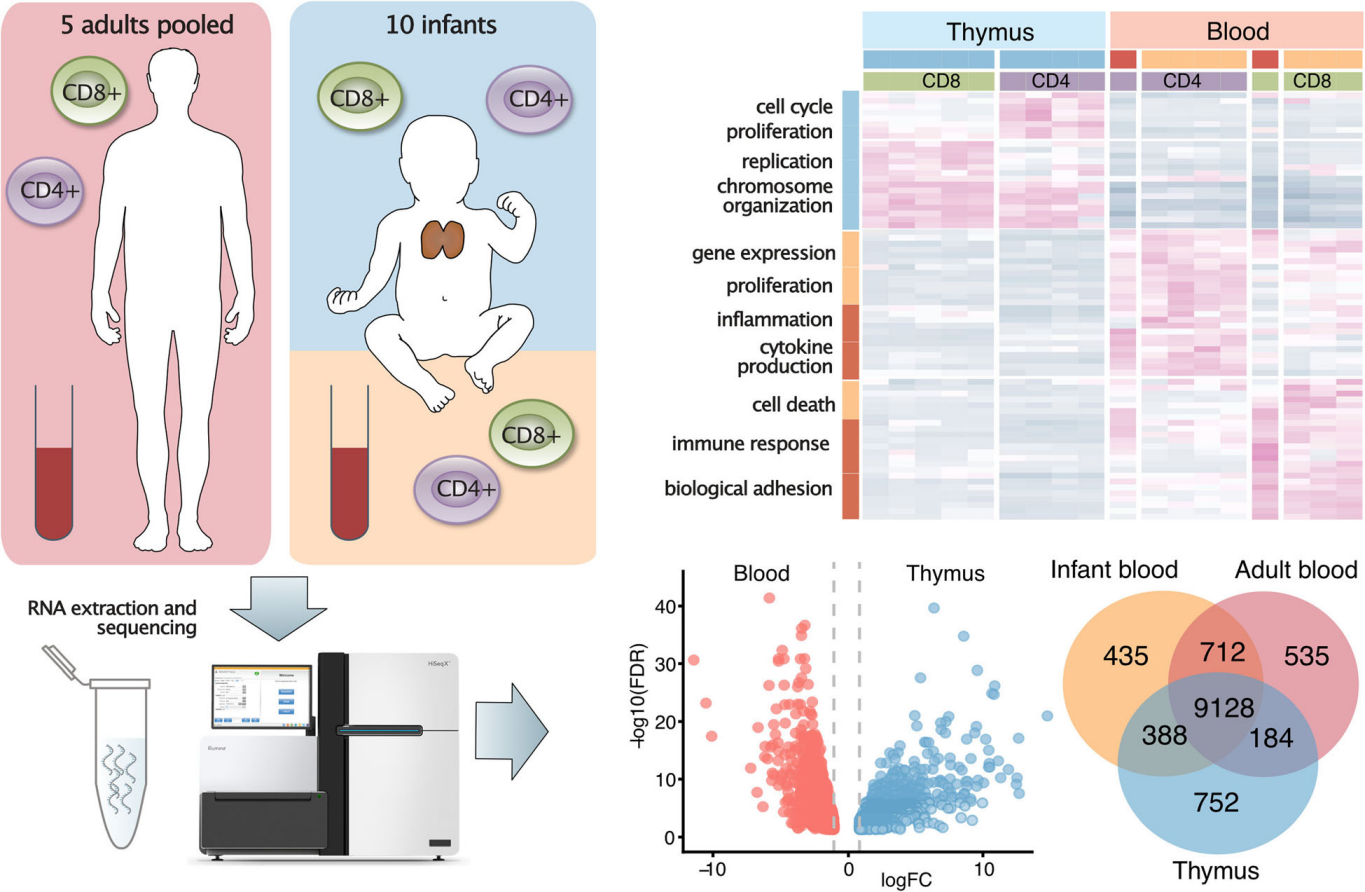
Graphical summary of the results. CD4+ and CD8+ T cells were isolated from infant blood and thymus, and a pool of adult blood samples. RNA was extracted and sequenced, before differential gene expression was assessed comparing the different tissues of origin, ages and cell populations. Differences in gene expression levels, number of uniquely expressed genes and enriched gene sets were detected

In this study, we present the transcriptomes from primary human CD4+ and CD8+ T cells from thymus and peripheral blood from young children and, in addition, provide comparisons to adult peripheral CD4+ and CD8+ T cells. A graphical summary of the results is displayed in Fig. 7. The transcriptomes deviated more according to site of origin, i.e. thymus vs blood, than according to T cell subtype or age. The thymic T cells showed the largest number of uniquely expressed genes, suggesting a more diverse transcription compared to peripheral blood derived T cells. CD4+ and CD8+ T cells showed more distinct differences in peripheral blood than in thymus, likely reflecting the differentiation and diversification of naïve T cells when encountering its cognate antigen in the periphery.

### T cell egress and migration

In mice, the T cell egress phenotype has been determined as Cd3 + Cd27 + Cd45ra + Cd62l + Cd69- [21]. In the thymus, *CD69* expression has been reported to be downregulated in mature SP thymocytes, enabling expression of *S1PR1* and egress from the thymus [22, 23]. We detected lower *CD69* expression in thymus compared to blood from infants, most pronounced in CD4+ T cells (Fig. 6d). CD69 and S1PR1 regulates the retention or egress of T cells from lymphoid tissue by forming a complex inhibiting the egress function of S1PR1, with little effect on the transcriptional level of *S1PR1* [24, 25]. In peripheral T cells, CD69 is an early activation marker [26, 27], where expression is rapidly and transiently induced following activation [28]. The high expression of both *CD69* and *S1PR1* in infant blood CD4+ T cells detected in this study, could suggest active recirculating of T cells between peripheral blood and lymphatic tissue of young children.

*PECAM1* (CD31) has been proposed as a marker for CD4+ recent thymic emigrants [29, 30]. The expression is down regulated upon proliferation after antigenic priming or homeostatic signals [31], in coherence with the high levels detected in thymic SP CD4+ T cells, compared to peripheral CD4+ T cells (Fig. 6d). In contrast, naïve CD8+ T cells egress the thymus expressing *PECAM1* and retain its expression during differentiation in the periphery [32]. In our study, the highest expression of *PECAM1* was detected in CD8+ infant blood T cells, followed by thymic CD8+ SP T cells. The overall expression of *PECAM1* was higher in CD8+ than CD4+ T cells, consistent with previously reported findings [10, 33].

In humans, the CD8+ recent thymic emigrant phenotype has been described as CD8 + CD103 + CD62L + CD27 + CD11a^dim^CD95^dim^ [34]. Homing to secondary lymphoid organs is enabled by CCR7 and CD62 ligand (CD62L/SELL), expressed on naïve T cells [35], in coherence with the high expression we observed in infant blood T cells. *CCR7* and *CD62* expression is high in central memory cells as well [36, 37], which could explain the high levels detected in adult peripheral blood CD4+ T cells, nearly as high as in infant blood. However, the recent article by Park et al. [38] identified KLF2 to be a regulator of thymic emigration. KLF2 is a transcription factor that regulates the expression of S1PR1 and CD62L [39]. Though not expressed in DP thymocytes, expression is induced in the mature SP thymocytes, both CD4+ and CD8+, and is maintained in naïve T cells until activation, introducing a rapid and profound loss of KFL2 [40]. We observed high expression of KLF2 in CD4+ and CD8+ T cells from both infant and adult blood, compared to thymus, indicating that the ratio of egressing or naive T cells was high in blood from both children and adults in both cell types.

### T cell differentiation

In our SP thymic T cells and in the infant blood CD8+ T, we detected expression of *BCL6*. BCL6 is essential for memory B cell development in germinal centers. In addition, follicular helper CD4+ (Tfh) cells are known to express this transcription factor [41]. CXCR5 is associated with B cell zone migration and homing, and has been well described in B cells and CD4 Tfh cells [42]. During unresolved infections or chronic inflammation, a subset of CD8+ cells localize to B cell follicles and differentiate to follicular CD8+ T cells, facilitated by the expression of *CXCR5* and *BCL6* [43, 44]. In a murine study, Bcl6 was identified as a key molecule for the establishment of memory CD8 T cells as well the peripheral CD8 T cell compartment in infancy [41]. Two decades ago, the BCL6 protein was detected in cortical thymocytes and some medullary thymocytes from human prenatal and postnatal thymi [40], supporting our findings of BCL6 expression in infant thymic T cells. All this supports our findings of the expression of BCL6 in CD8 T cells from both infant blood and thymic tissue, although further subtyping or single cell sequencing would elaborate their fate further.

In humans, six different isoforms of CD45 mRNAs have been isolated [45]. The majority of DP (> 90%) and SP (90%) thymocytes are CD45RO+, while egressing SP T cells are CD45RA+ [46, 47]. We find quite high expression of the CD45RO isoform in the thymic SP T cells, indicating what we have mainly captured SP T cells not yet ready for egress. Upon stimulation the naïve T cells lose their CD45RA and acquire CD45RO expression to become effector or memory cells, with a transitional stage of dual CD45RA/RO expression [48]. From our FACS data, we observed that a large proportion of the T cells in adults co-expressed CD45RA and CD45RO, which could suggest that a majority of the peripheral T cells were in a transitional stage.

### Distinct differences between infant and adult T cells

Amongst the top differentially expressed genes was the cytokine *CXCL8* (IL8), almost exclusively expressed in CD8+ infant blood T cells. CXCL8 is previously detected in human CD8+ from umbilical cord blood [10]. Elevated *CXCL8* expression in pre-term babies and umbilical cord blood compared to adult blood, indicates that T lymphocytes in very early life are intrinsically anti-inflammatory and also emphasizes qualitative distinctions between infants’ and adults’ immune systems [49]. Both CD4+ and CD8+ infant peripheral T cells displayed higher expression of CD44 than their adult blood counterparts. CD44 is upregulated after activation of naïve T cells and the elevated level is sustained for a while to protect against re-infection [50], and thereby also considered a marker for memory T cells in humans [51]. This suggests that the population of infant peripheral T cells are vigorously protecting the young body from previously unencountered invaders, and due to this high load of antigen exposure the memory T cells accumulates.

### Autoimmune diseases

Interestingly, about 3/4 of genes annotated to be involved in susceptibility to autoimmune diseases were found to be expressed in our T cell panel. Of these, more than 10% were not expressed in T cells from adult blood. This is noteworthy, as most studies addressing the expression of autoimmune risk genes investigate blood samples from adult individuals. An interesting instance is *SIRPG*, a gene associated with type 1 diabetes, which we have previously found to act an expression quantitative loci (eQTL) in human total thymic tissue [20]. Our current data revealed that *SIRPG* is particularly highly expressed the thymic CD8+ T cells, followed by the infant blood CD4+ and CD8+ T cells.

### Limitations of the study

Due to the young age of our participants, we were merely able to draw a 4 ml blood sample. Hence, the number of CD4+ and CD8+ T cells isolated from infant peripheral blood are lower (6 × 104–2.7 × 106) than the respective T cells isolated from infant thymi and adult peripheral blood (1.5 × 106–4.6 × 108). For the adult samples, we used a pool of 5 samples. This may have limited the number of transcripts detected in infant and adult peripheral blood T cells. Mature CD8+ T cells in human can express either the homodimer of CD8α-α or the heterodimer CD8α-β. Using a selection kit capturing the CD8alfa positive cells, enabled us to detect both dimers of CD8 T cells in our cohort. A pitfall of this choice, is that selected sub-population of human PBMC also express CD8α-α. Staining with CD3 in the CD8+ pool, we discovered that > 90% of the CD8+ cells are CD3+, hence considered T cells and also a minute proportion of NKT, while the remaining < 10% could be NK-cells, pDendrittic cells, Macrophages or monocytes, that may present CD8α on their surface under specific conditions. From the cell purity assessments, we uncovered lower purity of the CD8+ T cells isolated from infant blood than the CD8+ T cells from adult blood (75% vs 95% respectively). This may be since the kit is manufactured for adult human PBMC use. The impurities, particularly affecting the CD8+ T cells of infant blood, could have affected our results by adding gene transcripts originating from other cell types thereby influencing the assessed expression profiles of the infant blood. The low viability of our cells (63–88%) could indicate that the isolation procedure stressed the cells and thereby could also influence their observed expression profiles. Additionally, we have not distinguished naïve and memory T cells but their ratios are expected to differ between the T cell sources used in this study.

## Conclusion

This study provides novel insight into the transcriptome of the human primary SP T cells still residing in the thymus, and offers unique comparisons to primary blood derived T cells from infants and adults. Thymic T cells were enriched for gene ontology terms involved in cell proliferation and differentiation, when compared to infant blood derived T cells, whereas the infant blood T cells were enriched for immune responses, cell activation and signaling. We discovered that genes involved in migration, homing and recirculation, between peripheral blood and lymphatic tissue, were particularly active in infant blood T cells, suggesting active migration and recirculation in young children which likely also reflect the enrichment of naïve T cells. Genes encoding chemokine and interleukin receptors were particularly active in adult blood T cells, while upregulated genes in thymic T cells comprised genes involved in proliferation and early T cell development. From a list of 555 autoimmune disease associated genes, the majority were expressed in one or more T cell subset. However, ~ 11% were expressed in infant blood or infant thymic T cells alone, thus potentially evading detection in studies merely focusing on adult peripheral blood.

## Methods

### Sample material

Human thymic tissue was collected from 10 Caucasian infants (3 females and 7 males, age range: 5 days – 15 months), with no known syndromes, undergoing cardiac surgery to repair congenital abnormalities. From 5 of these infants (3 females, 2 males, age range: 5 days – 12 months), a 4 ml EDTA blood sample was collected. Furthermore, 27 ml blood was collected from 5 healthy adult individuals (ages 23–45, 3 females, 2 males). For the FACS study, 4 ml EDTA blood and thymic tissue was collected from a 6 years old male, while 10 ml EDTA blood was collected from two female adults (30 and 70 years old).

### Isolation of T cells from thymus and peripheral blood

Thymic tissue (~ 10 g) was collected and immediately washed in 10 ml PBS (Gibco, Thermo Fischer, MA, USA), before storage in a medium of 90% RPMI (Sigma-Aldrich, MO, USA) and 10% heat inactivated FCS (PAAlab, Pasching, Austria) for 30 min. The thymic tissue was further treated with Collagenase D (Roche Life Science, Basel, Switzerland) three times and Liberase TM (Roche Life Science) twice, until completely dissolved. Mononuclear cells were enriched from blood with Lymphoprep™ (Alere Technologies, Oslo, Norway) and EasySep tubes (STEMCELL Technologies, Vancouver, Canada) according to the manufacturer’s instructions. The PBMC of the 5 adults were pooled immediately prior to the cell sorting. We sorted the desired cell populations from homogenized single cell suspensions, manually, by targeted magnetic bead assays (STEMCELL Technologies, Vancouver, Canada). The CD4+ T cells from both thymus and blood were isolated with EasySep™ Human CD4 + CD25+ T Cell Isolation Kit (i.e. CD8-, CD14, CD16-, CD19-, CD20-, CD36-, CD56-, CD123-, TCRgamma/delta-, CD66b-, glycophorin A-, CD25-) to obtain single positive CD4 + CD25−/low. The blood CD8+ T cells were isolated using EasySep™ Positive CD8+ Selection Kit (STEMCELL Technologies), involving the monoclonal antibody clone RIV11, while the thymic CD8+ T cells were isolated by negative selection (CD4-, CD14-, CD16-, CD19-, CD20-, CD36-, CD56-, CD66b, CD123-, TCRgamma/delta-, glycophorin A-), using the human CD8+ T cell enrichment kit (STEMCELL Technologies). The pelleted cells were stored in RNAprotect® Cell Reagent (Qiagen, Hilde, Germany) at − 80 °C, before total RNA was extracted by RNeasy Plus mini Kit (Qiagen). According to manufacturer’s facultative suggestion, we used both the gDNA eliminator column, as well as DNase treatment. This provided a total of 4 thymic and 4 peripheral blood CD4+ CD25−/low T cell samples, and 5 thymic and 3 blood CD8+ T cell samples, in addition to two pools of blood derived CD4 + CD25- / low and CD8+ T cells, from 5 adults.

### Purity analyses of isolated T-cell subsets

To investigate the purity of the isolated cell populations, samples for flow cytometry were prepared from some of the thymi and blood samples in the project, as well as two blood samples from adult females (aged 30 and 70 years) and thymus and blood samples from a 6-year-old male for the CD45 RA/RO ratio. Samples for flow cytometry were analyzed on a BD Accuri C6 FCM Software (BD biosciences, New Jersey, USA), and we used Fluorescence Minus One Control to set the gates. To study the CD45 RA/RO ratio in the CD4+ T cell populations, we bead sorted the CD4 + CD25−/low by CD45RO+ selection, stained the two populations with either CD45RO-PE for CD45RO+ cells and CD45RA-APC for CD45RO- cells,

### Sequencing

The cDNA libraries were prepared using Truseq Stranded Total RNA Kit with Ribo-Zero GOLD set A (Illumina, California, USA # RS-122-2301). For the CD4+, and CD8+ thymic cells, sequencing was performed on Illumina HiSeq 2000 (Illumina, California, USA), 100 bp paired end, while the CD8+ blood T cells were sequenced on Illumina HiSeq 2500, 125 bp paired end.

### High performance computing

Computational analyses were performed using Services for Sensitive Data (TSD), a platform to store, analyze and share sensitive data provided by the University of Oslo, in compliance with the Norwegian “Personal Data Act” and “Health Research Act”.

### Read mapping and quantification

Low quality reads and adapter sequence was trimmed with Trimmomatic v0.33 [52, 53], using the following parameters: “ILLUMINACLIP:TruSeq3-PE.fa:2:30:10:8:true LEADING:3 TRAILING:3 SLIDINGWINDOW:4:15 MINLEN:36”, and PhiX sequence (used as spike-in all Illumina sequencing runs) was removed with BBMap v35.14 [53]. Reads were mapped with TopHat2 v2.1.0 [54] to both genome (EnsemblGRCh38) and transcriptome reference (Ensembl release 80), specifying estimated mate inner distance and mate standard deviation for each sample. Paired reads mapping in the right orientation to the exons were counted for each annotation gene using FeatureCounts (subread v1.4.6-p3) [55], with the following parameters: “–C –p –s 2 –t exon –g gene_id”.

### Selection of AID genes

The 555 genes associated with autoimmune diseases (AID) were selected from the National Institute of Health’s catalog of genome wide association studies (NHGRI) (http://www.ebi.ac.uk/gwas/). The following AID phenotypes were included in the search (November 2015): atopic dermatitis, ankylosing spondylitis, celiac disease, Crohn’s disease, ulcerative colitis, inflammatory bowel disease, juvenile idiopathic arthritis, multiple sclerosis, psoriasis, primary sclerosing cholangitis, rheumatoid arthritis, systemic sclerosis, type 1 diabetes. The selection was restricted to GWAS performed in Caucasian populations and annotated to SNPs with *P*-values < 5 × 10^− 8^. We did not include the X- or the Y-chromosome or the HLA-region.

### Differential expression analysis

Differential expression analysis was carried out in edgeR v3.16.5 [56]. TMM normalization was applied to account for compositional differences between libraries. Due to the complex multifactor design of the experiment, a generalized linear model (GLM) was used, considering the factors; cell type (CD4+, CD8+), tissue (thymus, blood) and age (infant, adult). Due to the large number of differentially expressed genes (DEGs) at FDR < 0.05; in total 14,975 unique DEGs, additional criteria; logFC> 1| < − 1 and logCPM> 1.5, was introduced to obtain biologically meaningful genes. The logCPM threshold of 1.5 was decided upon, due to its proximity to the local minimum of the bimodal logCPM density distribution (Supplementary Figure S8, Additional File 3). When determining the number of uniquely expressed and shared genes between the subsets, a cutoff of FPKM > 2 was used. To identify enriched biological processes, we used Gene Set Enrichment Analysis (http://software.broadinstitute.org/gsea) on significant DEGs from the pairwise comparisons. Redundant GO terms were reduced by REVIGO [57] web server tool. Genes associated with GO terms GO:0072676 lymphocyte migration and GGO:0030217 T cell differentiation were extracted from AmiGO v2.5.12 (http://amigo.geneontology.org/amigo), in addition to genes of special interest selected from the literature.

### De novo assembly of transfrags

To enable the detection of potential novel transcripts, guided de novo assembly was preformed using Stringtie v1.2.2 [58] (parameters: -B -G) with Ensembl GRCh38 release 80 annotation. The output gtf files were merged using cuffmerge (Cufflinks v2.2.1) [59], and the resulting merged gtf was provided as reference for a second Stringtie run (parameters -B -e -G). Assembled transfrags were normalized with edgeR’s Trimmed Mean of M (TMM). The coding potential was determined by the Coding Potential Assessment Tool (CPAT) [60]. Stringent filtering criteria was applied for length of transcript (> 200), length of ORF (< 120, > 100), FPKM expression level (multi-exon transfrags FPKM > 0.1 in > = 2 samples per group, single-exon transfrags FPKM > 1 in > = 2 samples per group) and coding potential (CPAT < 0.364, > 0.364, to classify transfrags as lncRNA or TUCP, respectively. Single-exon intergenic transfrags were not included. Using *blastn* (https://www.ncbi.nlm.nih.gov/blast/) against RefSeq release 98, February 2020, by the following criteria; identity > 90%, alignment length > 100, query or subject coverage > 80%, 72 tentative novel lncRNA and 47 TUCP transcripts were annotated. Under the following criteria; identity > 95%, alignment length > 200, query coverage > 80%, 6228 tentative novel isoforms were assigned to a known transcript.

## Additional files

**Additional File 1.** Purity plots of T cell suspensions.

**Additional File 2.** Supplementary Tables.

**Additional File 3.** Supplementary Figures.

## Data Availability

The datasets supporting the conclusions of this article are available in the Gene Expression Omnibus repository, Series accession number GSE139242 (https://www.ncbi.nlm.nih.gov/geo/query/acc.cgi?acc=GSE139242). EnsemblGRCh38, Ensembl release 80 was used as reference genome (ftp://ftp.ensembl.org/pub/release-80/fasta/homo_sapiens/dna/) and transcriptome (ftp://ftp.ensembl.org/pub/release-80/gtf/homo_sapiens). Unfortunately, we are not permitted to deposit the raw transcriptome data from this study. As required according to the Norwegian Health Research Act and the Norwegian Data Protection Act, we have a permit and approval from the Regional Ethical Committees to use and store Personal Data related to health. The RNA sequencing data for this study is Personal Data, as defined in Norwegian and European legislation. Even though all personal identifiers have been removed, the number of variables on the individual level is so extensive that identification of persons by use of other information from open sources is possible. Access to data is controlled and accepted by our Principal Investigator (PI), who has the formal responsibility as ‘Controller’ pursuant to Norwegian and European legislation. Sharing of data is a well-established routine for the PI, and after a Direct Transfer Agreement (DTA) has been signed and it has been approved by the ethical committee to submit data to a specific researcher or team, data will be shared. Data access can be requested directly from the PI at b.a.lie@medisin.uio.no or s.t.flam@medisin.uio.no.

## Abbreviations

AID: Autoimmune disease
BA: Blood adult
BI: Blood infant
CPAT: Coding Potential Assessment Tool
CPM: Counts per million
DEG: Differentially expressed gene
DP: Double positive
FPKM: Fragments Per Kilobase of transcript per Million mapped reads
lncRNA: Long non-coding RNA
MDS: Multidimensional scaling
ncRNA: Non-coding RNA
ORF: Open reading frame
PBMC: Peripheral blood mononuclear cell
RTE: Recent thymic emigrant
SP: Single positive
TI: Thymus infant
TMM: Trimmed mean of M
TUCP: Transcripts of uncertain coding potential
