# Supplementary material for: Transcriptome profiling of human thymic CD4+ and CD8+ T cells compared to primary peripheral T cells
Source: BMC Genomics. 2020 May 11;21:350. doi: 10.1186/s12864-020-6755-1 (PMC7216358; doi:10.1186/s12864-020-6755-1)
Supplement: Supplementary file 1 — Additional File 1. Purity plots of T cell suspensions. [file 12864_2020_6755_MOESM1_ESM.docx]

Additional file 1


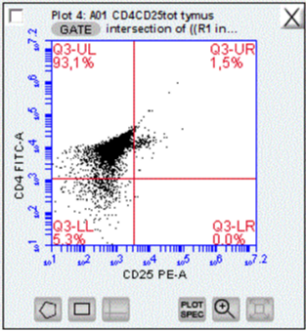

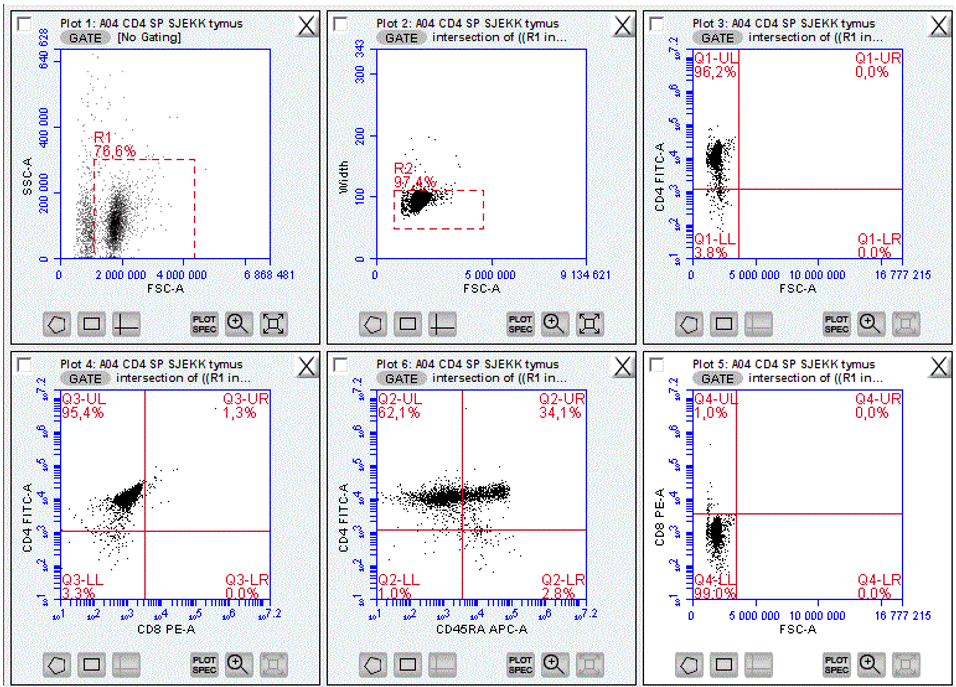


Figure S1. Purity of CD4+ SP T cells from thymus, assessed on BD Accuri C6. Gates are set using fluorescence minus one (FMO) control. The CD4+ T cells were isolated with EasySep™ Human CD4+CD25+ T Cell Isolation Kit.


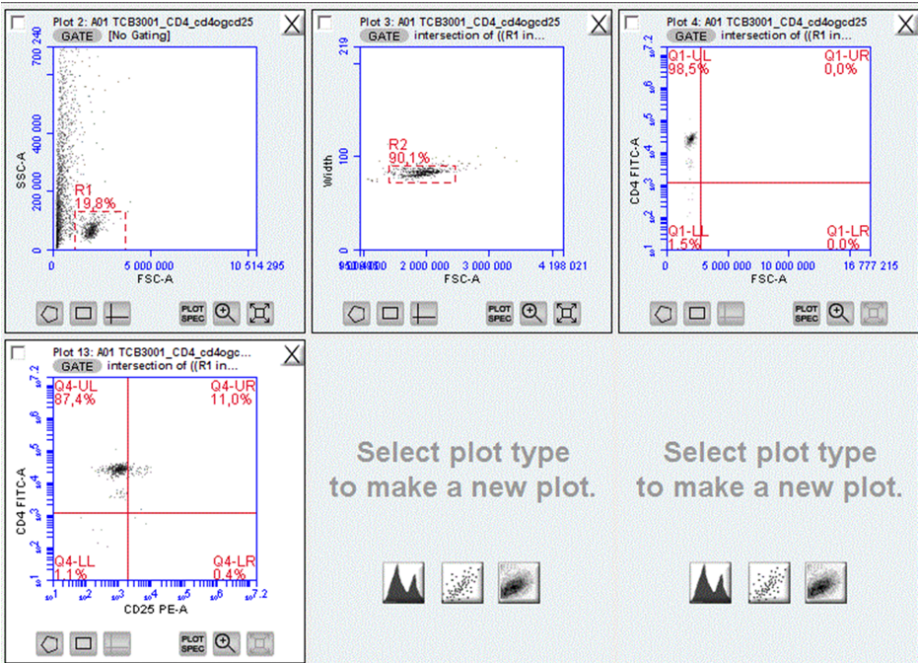


Figure S2. Purity of CD4+ SP T cells from infant blood, assessed on BD Accuri C6. Gates were set using FMO control. The CD4+ T cells were isolated with EasySep™ Human CD4+CD25+ T Cell Isolation Kit.


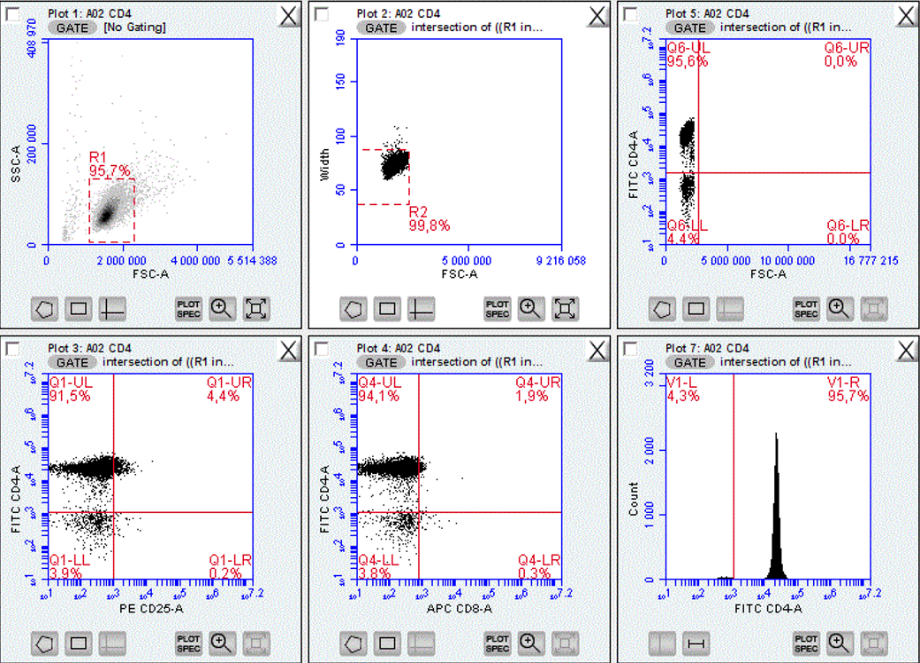


Figure S3. Purity of CD4+ SP T cells from adult blood, assessed on BD Accuri C6. Gates were set using FMO control. The CD4+ T cells were isolated with EasySep™ Human CD4+CD25+ T Cell Isolation Kit.


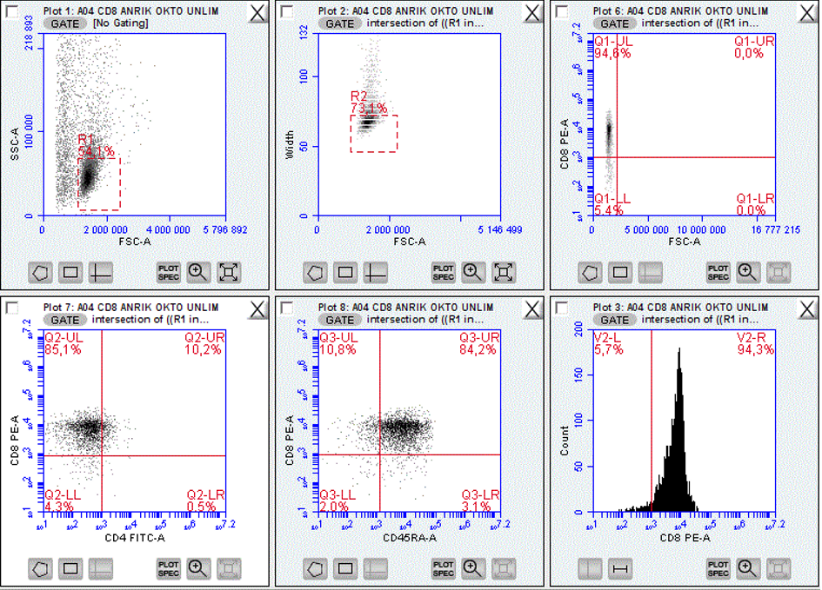


Figure S4. Purity of CD8+ cells from thymic tissue, assessed on BD Accuri C6. Gates were set using FMO control. The thymic CD8+ T cells were isolated by negative selection of CD4-, CD14-, CD16-, CD19-, CD20-, CD36-, CD56-, CD66b, CD123-, TCRgamma/delta- and glycophorin A-, using human CD8+ T cell enrichment kit from STEMCELL Technologies.


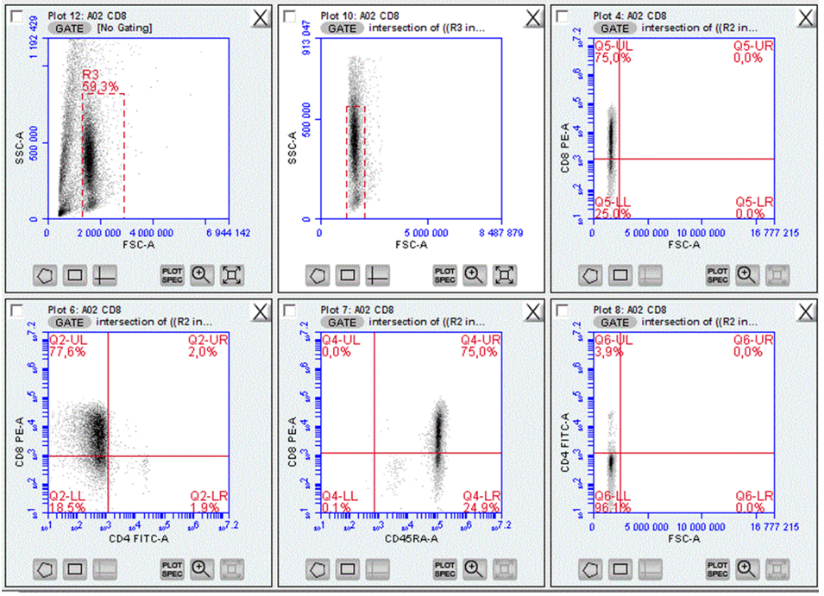


Figure S5. Purity of CD8+ cells from infant blood, assessed on BD Accuri C6. Gates were set using FMO control. The peripheral blood CD8+ T cells were isolated using EasySep™ Positive CD8+ Selection Kit from STEMCELL Technologies.


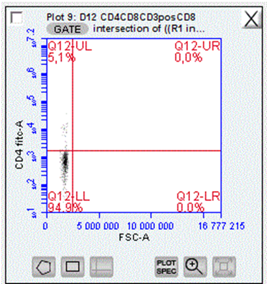

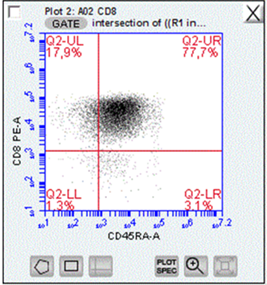

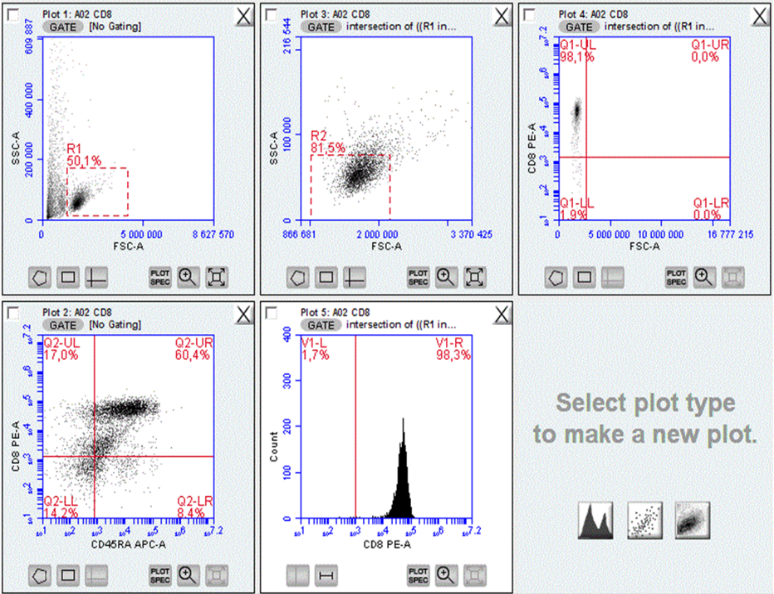


Figure S6. Purity of CD8+ cells from adult blood, assessed on BD Accuri C6. Gates were set using FMO control. The peripheral blood CD8+ T cells were isolated using EasySep™ Positive CD8+ Selection Kit from STEMCELL Technologies.


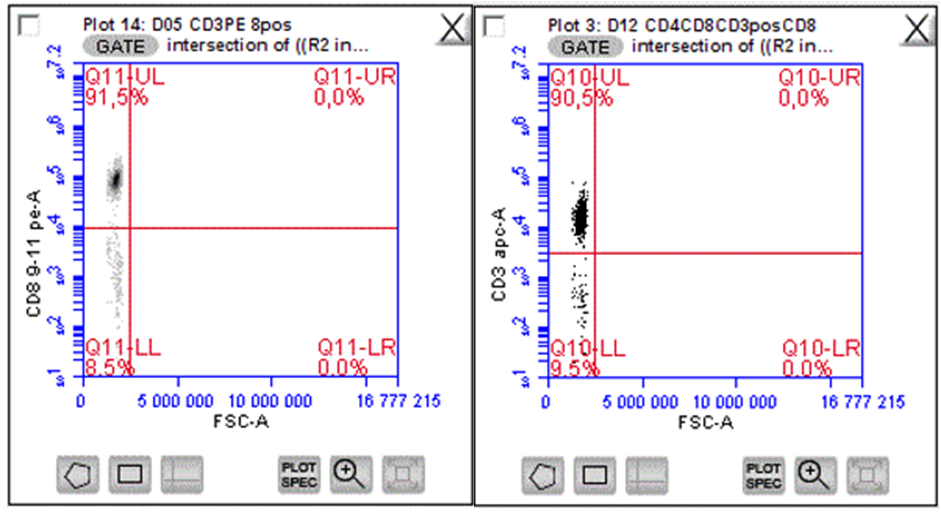


Figure S7. CD3+CD8alfa+ T cells in blood; the RIV11 clone, assessed on BD Accuri C6. Gates were set using FMO control.

Figure S8. CD4 thymic and peripheral T cells sorted by magnetic nanobeads. Flowcytometry analysis performed on a FACS Accuri, stained by CD45RO-FITC (clone UCHL1) and CD45RA-APC (clone HI100) and CD4 PE (clone OKT4) compensated by fluorescence minus one control.
