# Supplementary material for: Transcriptome profiling of human thymic CD4+ and CD8+ T cells compared to primary peripheral T cells
Source: BMC Genomics. 2020 May 11;21:350. doi: 10.1186/s12864-020-6755-1 (PMC7216358; doi:10.1186/s12864-020-6755-1)
Supplement: Supplementary file 3 — Additional File 3. Supplementary Figures. [file 12864_2020_6755_MOESM3_ESM.docx]

Supplementary Figures

Transcriptome profiling of human thymic CD4+ and CD8+ T cells compared to primary peripheral T cells

Hanna Helgeland^1,2*^, Ingvild Gabrielsen^1^, Helle Akselsen^1^, Arvind Y.M. Sundaram^1^, Siri Tennebø Flåm^1^, Benedicte Alexandra Lie^1*^

^1^Department of Medical Genetics, University of Oslo and Oslo University Hospital, 0450 Oslo, Norway

^2^Department of Radiation Biology, Oslo University Hospital, 0379 Oslo, Norway

*Address correspondence to Dr. Hanna Helgeland: Tel. +47-22781464, Email. hanna.helgeland@rr-research.no

Correspondence may also be addressed to Professor Benedicte Alexandra Lie: Tel. +47-23016646, Email. b.a.lie@medisin.uio.no

Supplementary Figure S1. A) number of exons B) coding probability score (not included for known transcripts) and C) transcript length density for known coding transcripts, tentative novel lncRNA, tentative novel alternative transcripts and transcripts of uncertain coding potential (TUCP), for all T cell subsets jointly. D) Complementary figure to Fig.2A, displaying log FPKM expression and number of transcripts discovered in thymic T cells, solely.


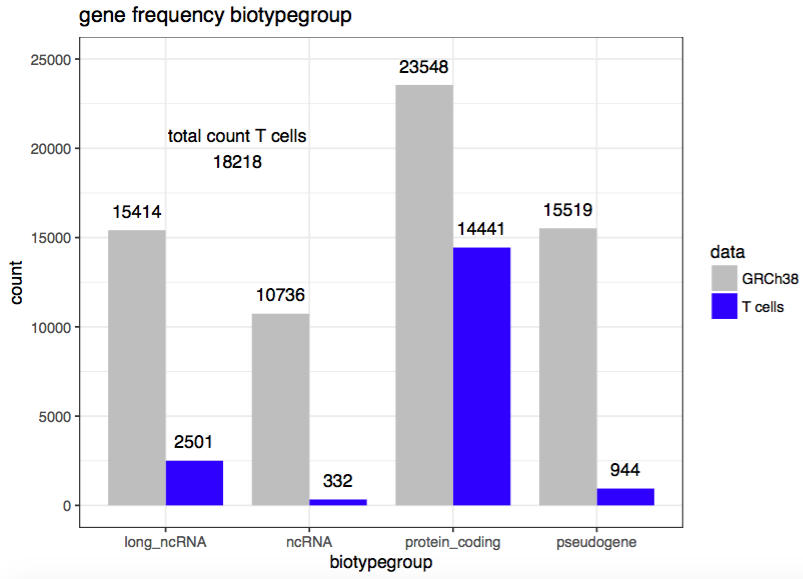


Supplementary Figure S2. Number of genes identified, after filtering low expressed genes (>1 pr mill count), grouped by biotype and compared to the used reference GRCh38.


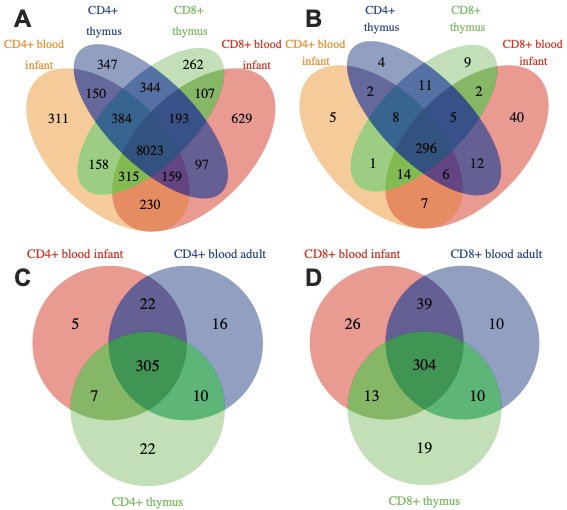


Supplementary Figure S3. Number of unique and commonly expressed genes (FPKM >= 2) between thymic and infant blood CD4+ and CD8+ T cells in A) all genes, and B) of in total 555 genes associated with AIDs, and in adult blood, infant blood and thymic C) CD4+ T cells, and D) CD8 + T cells.


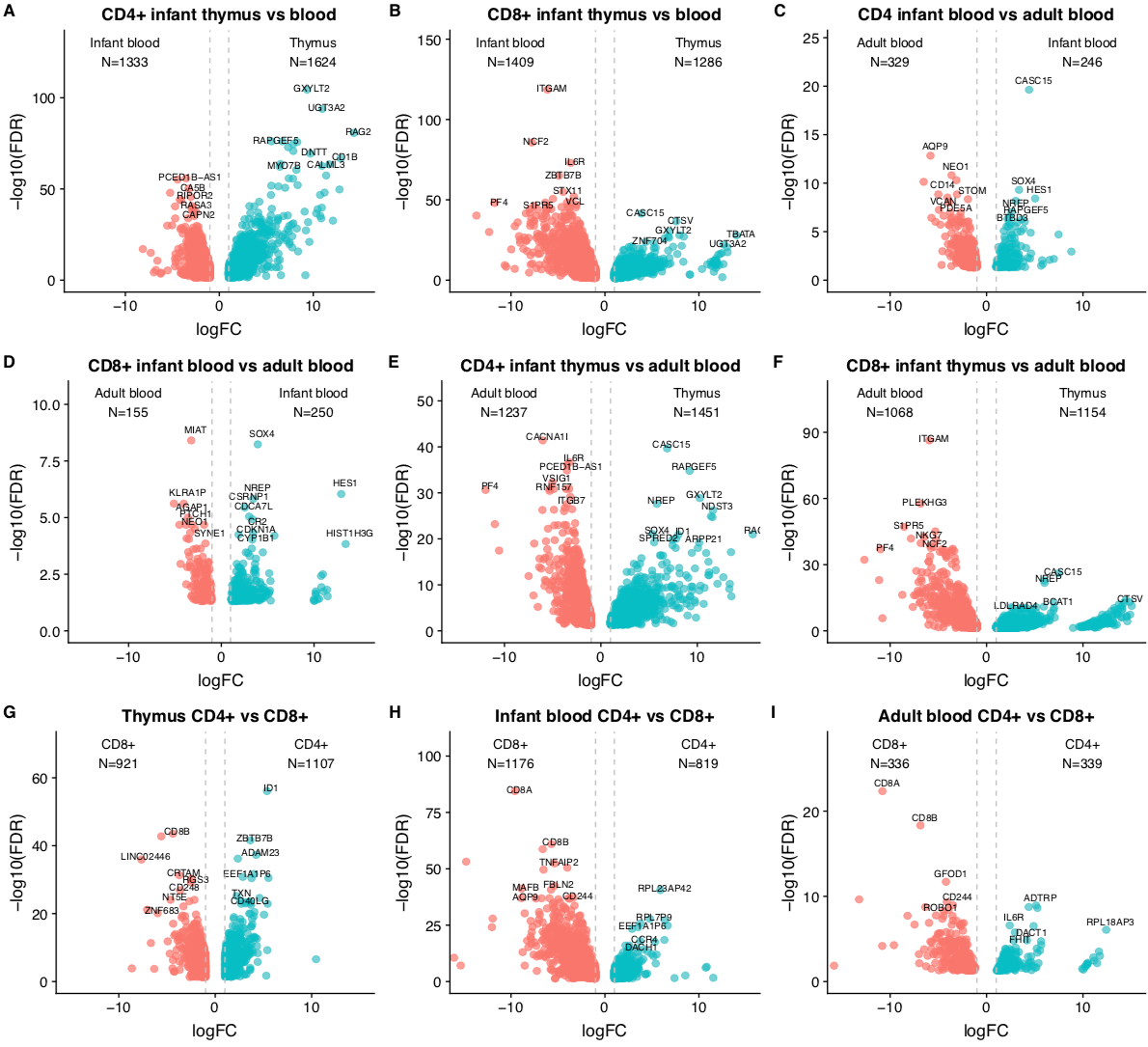


Supplementary Figure S4. Number of significant differentially expressed genes (FDR<0.05, logCPM>1.5, logFC>1) for the pairwise comparisons. Dotted vertical lines represent logFC 1 and -1.

Supplementary Figure S5. Clustering of the, in total, 5925 significant differentially expressed genes (FDR<0.05, logCPM>1.5, logFC>1) form the pairwise comparisons. Hierarchial clusterting method ward.D, euclidean distance measure.


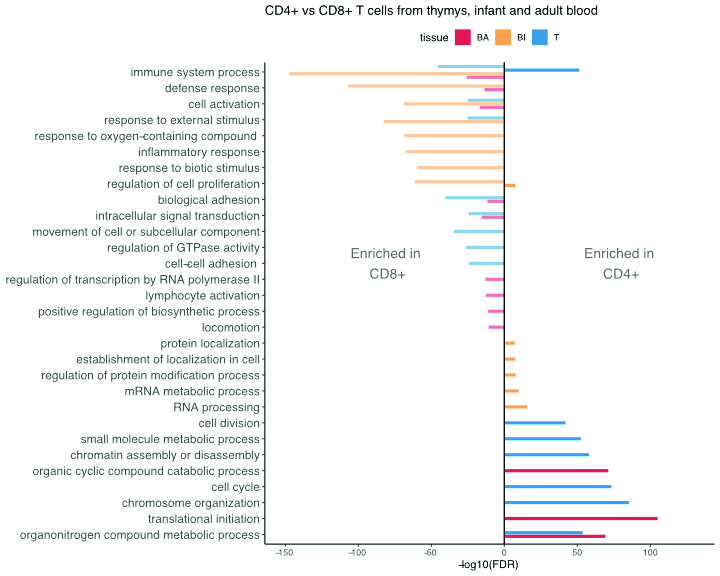


Supplementary Figure S6. Biological processes enriched in CD4+ T cells (positive values) vs CD8+ T cells (negative values) in thymus (blue), infant blood (orange) and adult blood (red). BA = blood adult, BI = blood infant, T = thymus infant.

Supplementary Figure S7. Gene expression of CD8B isoforms, for each T cell subset. ba = blood adult, bi = blood infant, ti = thymus infant.


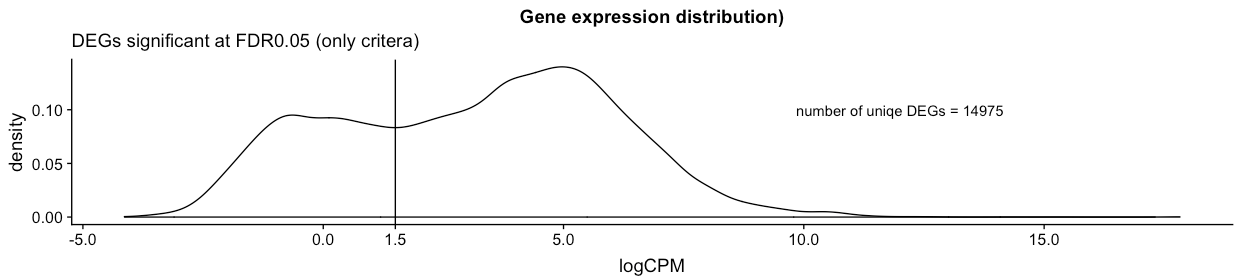


Supplementary Figure S8. LogCPM density distribution of significantly differentially expressed genes, at FDR<0.05.
